# Altered native stability is the dominant basis for susceptibility of α_1_-antitrypsin mutants to polymerization

**DOI:** 10.1042/BJ20131650

**Published:** 2014-04-25

**Authors:** James A. Irving, Imran Haq, Jennifer A. Dickens, Sarah V. Faull, David A. Lomas

**Affiliations:** *Cambridge Institute for Medical Research, Department of Medicine, University of Cambridge, Wellcome Trust/MRC Building, Hills Road, Cambridge CB2 0XY, U.K.; †Wolfson Institute for Biomedical Research, The Cruciform Building, University College London, Gower Street, London WC1E 6BT, U.K.

**Keywords:** cirrhosis, denaturation, disulfide, polymerization, serpin, stability, bis-ANS, 4,4′-dianilino-1,1′-binaphthyl-5,5′-disulfonic acid, HRP, horseradish peroxide, Ni-NTA, Ni^2+^-nitrilotriacetic acid, RCL, reactive centre loop, TMAO, trimethylamine *N*-oxide

## Abstract

Serpins are protease inhibitors whose most stable state is achieved upon transition of a central 5-stranded β-sheet to a 6-stranded form. Mutations, low pH, denaturants and elevated temperatures promote this transition, which can result in a growing polymer chain of inactive molecules. Different types of polymer are possible, but, experimentally only heat has been shown to generate polymers *in vitro* consistent with *ex vivo* pathological specimens. Many mutations that alter the rate of heat-induced polymerization have been described, but interpretation is problematic because discrimination is lacking between the effect of global changes in native stability and specific effects on structural mechanism. We show that the temperature midpoint (*T*_m_) of thermal denaturation reflects the transition of α_1_-antitrypsin to the polymerization intermediate, and determine the relationship with fixed-temperature polymerization half-times (*t*_0.5_) in the presence of stabilizing additives [TMAO (trimethylamine *N*-oxide), sucrose and sodium sulfate], point mutations and disulfide bonds. Combined with a retrospective analysis of 31 mutants characterized in the literature, the results of the present study show that global changes to native state stability are the predominant basis for the effects of mutations and osmolytes on heat-induced polymerization, summarized by the equation: ln(*t*_0.5,mutant_/*t*_0.5,wild-type_)=0.34×Δ*T*_m_. It is deviations from this relationship that hold key information about the polymerization process.

## Abstract 2

Mutations in α_1_-antitrypsin increase or decrease its tendency to form pathogenic ordered polymer chains. The present study shows that these effects are primarily exerted through changes in native state stability, and seldom through direct effects on the polymerization mechanism.

## INTRODUCTION

Serpins are globular proteins that predominantly act as protease inhibitors in a wide range of proteolytic cascades and environments. They are found ubiquitously in eukaryotes and in some bacterial and archaeal genomes [1,2]. The serpin native state presents an exception to Anfinsen's principle [3] as it is not the most stable form of the protein; it is a kinetically trapped intermediate whose conversion into the most stable state occurs upon proteolytic cleavage of an exposed RCL (reactive centre loop). This is the basis of the serpin inhibitory mechanism [4]. Various point mutations can perturb the barrier between native and stable states, leading to the adoption of alternative inactive forms of the serpin [5]. One such conformer is the serpin polymer, which is an ordered aggregate linearly propagated via an intermolecular linkage with a characteristic ‘beads-on-a-string’ appearance when visualized by EM [6]. This linkage is extremely stable: polymers can readily be extracted intact from patient samples [7], requiring mechanical disruption in the case of dense inclusions [6], and are resistant to dissociation by urea [8,9].

Serpin polymers result in a group of diseases termed the serpinopathies [10]. Many serpinopathies are the consequence of a loss of inhibitory activity, whereas others are caused by a toxic gain-of-function from the accumulation of serpin aggregates in the cell of synthesis. In α_1_-antitrypsin deficiency, intracellular retention of ordered aggregates causes neonatal hepatitis and cirrhosis, and a decreased concentration of protein in the plasma underlies early-onset emphysema [11,12]. Similarly, retention of point mutants of neuroserpin within neurons results in an autosomal dominant dementia [13]; whereas the polymerization of α_1_-antichymotrypsin, C1-inhibitor and anti-thrombin cause plasma deficiency of these proteins in association with emphysema, angio-oedema and thrombosis respectively [14].

Serpin polymerization can also occur under destabilizing conditions, such as at low pH [15] and in the presence of detergent [8]. In particular, induction of α_1_-antitrypsin polymerization *in vitro* by the addition of chemical denaturants or through an increase in temperature has been a staple experimental technique for more than two decades [6,8]. However, biophysical [4,6,16–20] and crystallographic [21,22] studies have provided evidence that these approaches yield polymers of markedly different structural character. The stability of α_1_-antitrypsin has been extensively characterized in the presence of a chemical denaturant [19,23,24], and concentrations at which the unfolding intermediate ensemble is most populated coincides with a tendency to polymerize [25]. Although there are probably commonalities between the intermediate states under various destabilizing conditions, notably only heat-generated material has been demonstrated to share a cryptic epitope with *ex vivo* pathological specimens [26,27]. For this reason, heat was chosen as the inducer for the experiments described in the present paper.

The midpoint of thermal denaturation (*T*_m_) is one measure of the thermal stability of a protein. Analyses of several mutants have suggested an inverse trend occurs between the *T*_m_ and rate of polymerization [28,29]. In the former work, we hypothesized that this correlation related to a specific functional role played by these mutants in the opening of β-sheet A; in the latter, it was a marked departure in behaviour that was interpreted as mechanism-specific. This underlines a problem in the interpretation of polymerization rate data. Further, several studies have drawn inferences regarding polymerization mechanism from introduced disulfides [21,22,30,31], despite the ability of disulfide bonds to stabilize a native fold by reducing local secondary structure mobility [32]. Ideally, it would be possible to decouple the effect on native state stability from the effect on mechanism. However, the extent to which native stability itself plays a role, as opposed to specific mechanistic perturbations, has not yet been demonstrated.

We undertook a detailed analysis of the effect of increased global stability of the native fold on the propensity to polymerize. Compounds known to globally stabilize serpins, sodium sulfate, sucrose and TMAO (trimethylamine *N*-oxide) [33–35], were characterized for their ability to alter both the midpoint of thermal denaturation and the polymerization half-time (*t*_0.5_) of α_1_-antitrypsin. It was found that increased thermal stability shared a strong direct correlation with a reduced rate of polymerization. This relationship was confirmed by a range of engineered disulfides and two point mutants of α_1_-antitrypsin. Further, on the basis of our previous observation that polymer formation follows an apparent Arrhenius-type temperature dependence [36], we used a simple normalization to account for the use of different experimental temperatures, and applied it to data from five published studies. The collective results demonstrate that changes in stability of the native state, and not specific interference with molecular mechanism, are the dominant cause of mutant-based effects on polymerization.

## EXPERIMENTAL

### Reagents

Unless otherwise stated, reagents were obtained from Sigma, Alfa Aesar or MP Biomedicals, and numerical analyses were performed using Prism (GraphPad) and COPASI [37]. Expression media was from Formedium.

### Plasmid generation for the expression of recombinant α_1_-antitrypsin *in vitro*

The pQE-30 and pQE-81L plasmids (Qiagen) containing wild-type (M) α_1_-antitrypsin ORF with the C232S mutation (AT_C232S_) were used to express recombinant α_1_-antitrypsin [38]. The C232S substitution obviates the need for a reducing agent in the assay buffer and was used as the control for *in vitro* experiments; this variant has been found to behave in an equivalent fashion to wild-type protein in previous studies (for example [16,36]). Sequential mutagenesis steps were used against the AT_C232S_ background to generate the novel double-cysteine residue mutants E162C/V170C and K191C/T339C, as well as K168C/F189C, S283C/P361C and S292C/T339C that have been reported previously [21,30,31]. The point mutants K331V [29] and K335A [39] were also generated.

### Expression and purification of recombinant α_1_-antitrypsin

Plasmids containing AT_C232S_ and mutants of α_1_-antitrypsin on the C232S background were transformed into SG13009/pREP4 cells and BL21(DE3) cells (Novagen) for pQE-30 and pQE-81L-based constructs respectively. Recombinant proteins were expressed and purified as described previously [38], before buffer exchange into 20 mM Tris/HCl and 100 mM NaCl (pH 7.4) and storage at −80°C. The resulting proteins were assessed by SDS/PAGE (4–12% gel), CD, thermal stability and for their ability to inhibit bovine α-chymotrypsin. The double-cysteine variants were found to be predominantly in the disulfide-bonded form (>90%) by SDS/PAGE, and by their relative inability to form adducts with thiol-reactive PEG5K/maleimide and dithiodipyridine [40]. The CD spectra were essentially identical in shape, indicating no discernible effect on the structure of α_1_-antitrypsin (Supplementary Figure S1 at http://www.biochemj.org/bj/460/bj4600103add.htm).

### CD analysis

Far-UV spectra were obtained using a JASCO J-810 spectrapolarimeter and a 0.1-mm pathlength cell. Samples were dialysed into 10 mM NaH_2_PO_4_/Na_2_HPO_4_ (pH 7.4) and adjusted to 0.5 mg/ml before analysis, and spectra were recorded by scanning from 260 to 180 nm at a rate of 50 nm/min and averaging the replicate curves. Thermal denaturation experiments made use of a 2-mm pathlength cell, or stirred 5-mm pathlength, cuvette, with measurement of the CD signal at 225 nm and a linear increase in temperature at a rate of 1°C or 5°C/min. A thermistor probe immersed in the sample was used to directly monitor the temperature change, and the temperature profile reported by the software was adjusted as appropriate. This was of particular necessity at the higher temperature gradient.

### Fluorescence-based thermal denaturation assay

The stability of α_1_-antitrypsin was investigated by thermal denaturation in the presence of a 5× concentration of SYPRO Orange dye solution (Life Technologies) in 25 mM NaH_2_PO_4_/Na_2_HPO_4_ and 75 mM NaCl (pH 7.4), at a final protein concentration of 0.025–0.1 mg/ml and a 20 μl volume [41]. Protein samples were heated from 25°C to 95°C at a rate of 1–5°C/min using an Applied Biosystems 7500 quantitative real-time PCR instrument (Life Technologies). A curve describing a two-state unfolding transition [42] was fitted to the data by non-linear regression for the calculation of the transition midpoint temperatures (*T*_m_).

### Densitometry of α_1_-antitrypsin polymers

Following polymerization, α_1_-antitrypsin was subjected to non-denaturing PAGE electrophoresis using a 4–12% (w/v) acrylamide Bis-Tris gel (Life Technologies). After staining with Coomassie Blue, the gel was digitized and densitometric analysis performed using GelAnalyzer 2010a software (http://www.gelanalyzer.com/). This software was also used to process the gel images presented by Gilis et al. [29].

### Polymerization kinetics

An assay was previously developed in our laboratory that follows the rate of polymer formation by monitoring FRET be-tween Ni-NTA (Ni^2+^-nitrilotriacetic acid)-conjugated Atto-550 and Atto-647N fluorescent probes [36]. α_1_-Antitrypsin was diluted to 0.2 mg/ml in 10 mM NaH_2_PO_4_/Na_2_HPO_4_ and 100 mM NaCl (pH 7.4) containing 4 μM Atto550–Ni-NTA and 4 μM Atto647N–Ni-NTA in a total volume of 20 μl. Reactions were followed for 8–16 h at 55°C, 60°C and 65°C on an Applied Biosystems 7900HT quantitative PCR instrument. FRET efficiency was calculated as the ratio between the mean of the 655–660 nm and 540–590 nm bins, and expressed as a multiple of the value recorded at the start of the experiment. Polymerization has been proposed to proceed via a simple reaction pathway, in which a monomer (M) becomes activated (M*) before the formation of polymers (P) [28]:

Mk1↔M*M*+M*k2→P

although there is evidence that the process involves more than one intermediate state [36] and, at least for denaturant-activated material, dimers polymerize more rapidly than monomers [44]. Rates of unfolding can often be conveniently represented as the half-time of the conformational transition *t*_0.5_ [45], which is related to the first-order rate constant *t*_0.5_=0.693/*k*_obs_. We made use of this property in order to minimize assumptions and avoid model bias that could potentially arise from fitting parameters to one reaction scheme over another. A two-phase exponential function could be fitted satisfactorily to FRET progress curves by non-linear least-squares regression in COPASI [37]. The time to half-maximal signal was then determined numerically from the curve using a script in GNU Octave (https://www.gnu.org/software/octave/).

### Western blot analysis of polymers using the 2C1 antibody

Recombinant AT_C232S_ or mutant α_1_-antitrypsin was heated for 8 h in 20 μl reaction volumes at various temperatures in a thermal cycler. On completion, samples were snap-frozen in liquid nitrogen. Defrosted samples were resolved by non-denaturing PAGE, and subjected to Western blot analysis with binding of the 2C1 mouse monoclonal antibody overnight at 4°C [26]. HRP (horseradish peroxide)-conjugated anti-mouse antibody (1:10000 dilution; Sigma) was used as the secondary antibody. After developing, the membrane was stripped using 0.2 M NaOH for 10 min, re-blocked and incubated with rabbit polyclonal antibody (from Professor Juan Pérez, University of Málaga, Málaga, Spain), which recognizes total α_1_-antitrypsin, at a 1:4000 dilution overnight at 4°C. HRP-conjugated anti-rabbit antibody (1:10000 dilution; Sigma) was used as the secondary antibody. Blots were visualized using the SuperSignal West Pico chemiluminescent substrate (Thermo Scientific).

### Inhibitory activity of α_1_-antitrypsin and variants

Bovine α-chymotrypsin was titrated using *p*-nitrophenyl acetate [47]. The stoichiometry of inhibition of AT_C232S_ and mutants of α_1_-antitrypsin was determined by incubation with 0.5 μM bovine α-chymotrypsin for 30 min at room temperature (21°C) in 20 μl of assay buffer [20 mM Tris/HCl, 0.1 M NaCl, 0.1% PEG 8000 and 10 mM CaCl_2_ (pH 8.0)]. N-succinyl-Ala-Ala-Pro-Phe-p-nitroanilide substrate (180 μl of 200 μM) was added and the absorbance at 405 nm recorded for 5 min using a ThermoMax plate reader (Molecular Devices). Linear regression was used to calculate the ratio of inhibitor required to completely abrogate enzyme activity. The association rate constant of inhibitor with enzyme (*k*_ass_) was measured by reaction progress curves under pseudo-first-order conditions for 4 h at 30°C with a final concentration of 5–600 nM inhibitor, 200 μM substrate and 0.5 nM bovine α-chymotrypsin. Data analysis was performed as described previously [48].

### Polymerization kinetics described in the literature

Polymerization rates were obtained from studies reported in the literature in which values had been determined from the loss of monomer as observed by native gel densitometry [29,49–51] and using intrinsic tryptophan fluorescence [28]. In the case of gel images presented by Gilis et al. [29], densitometry was performed retrospectively using GelAnalyzer 2010a software (http:://gelanalyzer.com). One study used both gels and bis-ANS (4,4′-dianilino-1,1′-binaphthyl-5,5′-disulfonic acid) dye to monitor the polymerization reaction [50]. Interestingly, an examination of the rates they obtained reveals the closest linear correspondence between gel densitometry and the *k*_cc_ value, the ‘rate of conformational change’ calculated from the rapid initial increase in bis-ANS fluorescence (Supplementary Figure S2 at http://www.biochemj.org/bj/460/bj4600103add.htm). Thus the *k*_cc_ values presented by Cabrita et al. [52] using bis-ANS, rather than the *k*_agg_ values, were used as a basis for comparison (but were not included in the data used to derive the *t*_0.5_–*T*_m_ relationship).

## RESULTS AND DISCUSSION

Characterization of the pathway that underlies the serpin polymerization process is important, as it has direct relevance to toxic gain-of-function and loss-of-function phenotypes in affected individuals. Many variants of α_1_-antitrypsin have been described that result in an altered tendency to polymerize [14]. To interrogate these mutants, polymerization can be induced *in vitro* by the addition of chemical denaturant or at elevated temperatures [6,8], and the production of oligomers followed by non-denaturing PAGE or spectroscopic methods such as ANS binding, tryptophan fluorescence, CD and FRET [25,28,36]. Effects on serpin stability have typically been assessed using related approaches, such as equilibrium unfolding in chemical denaturant [23] and thermal unfolding experiments [42], over a range of destabilizing conditions. It has been observed qualitatively, in different studies using disparate methodologies, that the rate of polymerization appears inversely related to the stability of the native state [28,29,33–35]. We sought to characterize this relationship in detail, at several temperatures, in an attempt to distinguish the specific effect of mutations on structural mechanism from non-specific effects on native state stability.

### Thermal stability assays report the transition to a polymerization intermediate, not a globally unfolded state

Thermal denaturation experiments, using CD or environment-sensitive fluorescent dyes, such as SYPRO Orange, give information about the native stability of a protein with respect to the unfolded state [53,54], represented at its most basic by a transition midpoint temperature *T*_m_. In contrast with the double-transition observed with denaturant-induced unfolding [23], CD-based thermal unfolding analyses of PAI-1 (plasminogen-activator inhibitor-1) [42] and α_1_-antitrypsin [28] report a single transition. It may be that there is only a poorly populated thermal unfolding intermediate; alternatively, the change reported is due to other than a fully denatured state. It is noteworthy that in constant-temperature experiments, a change in CD signal is observed that precedes polymerization [36]. To characterize the transition that is reported by CD during thermal unfolding, we performed experiments using recombinant wild-type α_1_-antitrypsin (AT_WT_), and a RCL double mutant with a comparable thermal stability that polymerizes more slowly due to a greater transition-state barrier to polymer formation (L353D/A355D; denoted as AT_P64_) [36].

**Figure 1:**
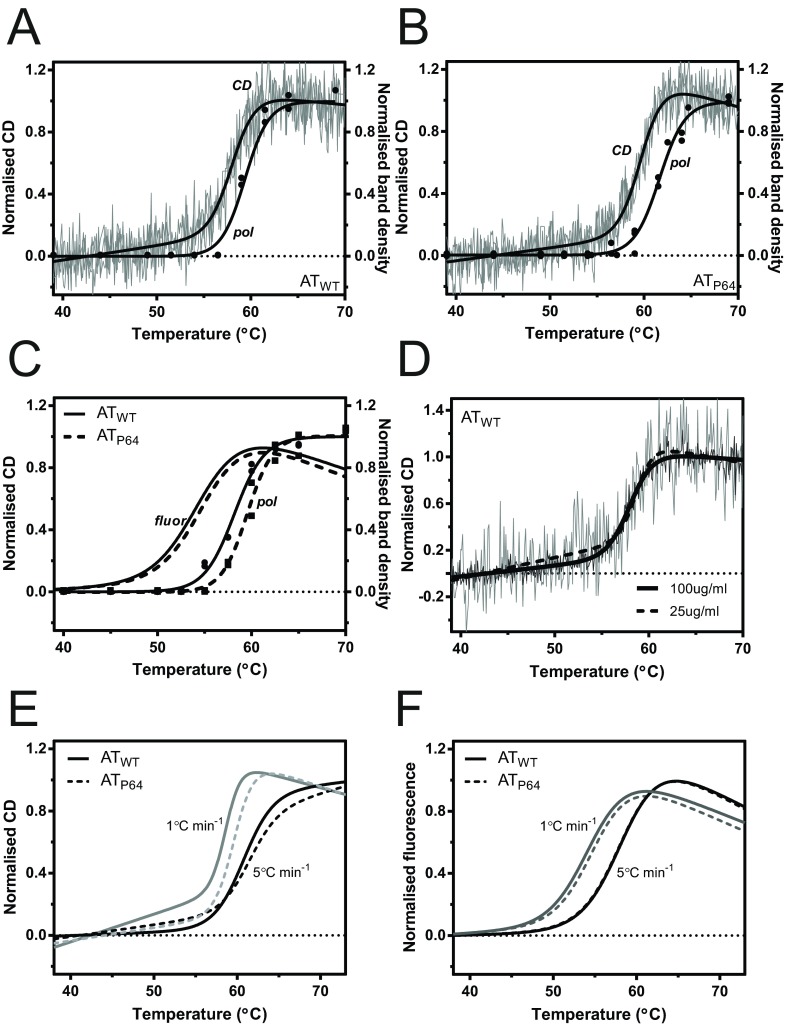
Characterization of thermal denaturation experiments with α_1_-antitrypsin (**A**) Recombinant wild-type α_1_-antitrypsin (AT_WT_) was heated at a constant rate of 1°C/min from 25°C to 95°C with continuous measurement of CD at 225 nm (grey lines). Periodically, aliquots were removed, analysed by non-denaturing PAGE (an example is shown in Supplementary Figure S3A at http://www.biochemj.org/bj/460/bj4600103add.htm), and the amount of polymer determined using densitometry (●). The result of non-linear regression analysis of the normalized data for two independent experiments, using an equation describing two-state unfolding [42], is shown as solid curves labelled ‘CD’ and ‘pol’. (**B**) Identical experiments were repeated for a L353D/A355D mutant (AT_P64_). (**C**) Summary of thermal unfolding experiments as monitored by an increase in the fluorescence of SYPRO Orange for AT_WT_ (solid lines and ●) and AT_P64_ (broken lines and ■) as the sample is heated at 1°C/min. Periodically, aliquots were removed and analysed by non-denaturing PAGE for the amount of polymer (● and ■). Lines show fits of an equation describing two-state unfolding. (**D**) The normalized CD data from thermal unfolding experiments of two concentrations of AT_WT_ (25 and 100 μg/ml) at a constant rate of 1°C/min are shown. The thick lines indicate the fit of an equation describing two-state unfolding. (**E**) AT_WT_ (solid lines) and AT_P64_ (broken lines) were heated at 1°C/min (grey lines) and 5°C/min (black lines), with unfolding monitored by the change in CD at 225 nm. Lines show the curve fit to three independent experiments. (**F**) AT_WT_ (solid lines) and AT_P64_ (broken lines) were heated at 1°C/min (grey lines) and 5°C/min (black lines), with unfolding monitored by the change in SYPRO Orange fluorescence. Lines show the curve fit to three independent experiments.

For initial unfolding experiments, the temperature of each sample was increased at a rate of 1°C/min with continuous measurement of CD at 225 nm, and aliquots were periodically removed from the cuvette for analysis by non-denaturing PAGE and densitometry. A comparison of the non-denaturing gel and CD progress curve for AT_WT_ shows that the change in CD signal precedes the appearance of polymer on the gel, with a calculated midpoint difference of 1.4±0.4°C (Figure 1A, Supplementary Figure S3A at http://www.biochemj.org/bj/460/bj4600103add.htm, and Table 1). As α_1_-antitrypsin polymers are known to be extremely stable and do not dissociate even in the presence of high concentrations of urea [9,55], this disparity is not the result of dissociation during electrophoresis. Indeed, for the AT_P64_ mutant, the appearance of polymer was further delayed on the gel, with a polymer midpoint 1.9±0.4°C greater than the CD transition (Figure 1B, Supplementary Figure S3A and Table 1). As AT_P64_ has been shown to exhibit an increased energetic barrier late in the polymerization pathway [36], this is consistent with CD reporting foremost an early activation step that precedes polymerization. The thermal melt was also repeated using SYPRO Orange, a dye whose fluorescent quantum yield increases substantially in a non-polar environment, and has been used previously with α_1_-antitrypsin [36]. Again, the increase in fluorescence preceded polymer formation, in this case by an increased margin of 3.2±0.5°C and 4.4±0.7°C for the wild-type and AT_P64_ mutant respectively (Figure 1C and Table 1).

**Table 1:** Transition midpoint temperatures (*T*_m_) of AT_WT_ and AT_P64_ calculated from thermal denaturation curves Samples were monitored for changes in CD, density of polymer bands resolved by non-denaturing PAGE, and SYPRO Orange fluorescence (Dye) during heating at a fixed rate. Curves describing two-state unfolding [42] were fit to the data from two to four independent experiments. Individual curve midpoints±S.D. are shown.

			*T*_m_ (°C)	
Reporter	Concentration (μg/ml)	Heating rate (°C/min)	AT_WT_	AT_P64_
CD	100	1	58.1±0.2	59.8±0.1
PAGE	100	1	59.5±0.3	61.7±0.4
CD	25	1	58.5±0.5	–
CD	25	5	60.9±0.2	61.3±0.6
Dye	100	1	55.0±0.5	55.2±0.5
PAGE (+dye)	100	1	58.2±0.1	59.5±0.5
Dye	100	5	58.5±0.1	58.5±0.1

Unfolding is a first-order process, whereas polymerization is concentration-dependent. When the CD denaturation assay was repeated at a 4-fold lower concentration, an almost identical value for *T*_m_ was obtained (Figure 1D and Table 1), as was the case when the SYPRO Orange reporter was used (Supplementary Figure S3C and Table 1). This insensitivity to concentration is consistent with first-order behaviour. However, different midpoints were obtained by both methods when the experiments were repeated at a higher heating rate of 5°C/min (Figures 1E and 1F, and Supplementary S3B), indicative of an unfolding process under kinetic, rather than thermodynamic, control. Such a scenario can arise even when the recorded change reflects a reversible process, if there is competition from a subsequent kinetically controlled step [56]. The association of monomers to form polymers, whose stability against dissociation has long been recognized [8,55,57], certainly would be an example of this.

Correspondingly, conditions under which intermolecular association would be expected to play a less prominent role show convergence of CD-reported *T*_m_ values for AT_WT_ and AT_P64_: a difference of 0.4°C at 25 μg/ml and 5°C/min compared with 1.7°C at 100 μg/ml and 1°C/min (Table 1). Indeed, when AT_WT_ was subjected to thermal unfolding up to the transition midpoint temperature, and subsequently cooled, the CD measurement returned to an intermediate value, suggesting a partially reversible unfolding event (Supplementary Figure S4 at http://www.biochemj.org/bj/460/bj4600103add.htm).

The detectable separation of the spectroscopic changes from polymerization indicates that the branch of the pathway that yields polymers is rate-limiting. Thus it is apparent that these two common methods of evaluating thermal stability report the transition to a thermal polymerization intermediate state, in a manner that is influenced by, but not contingent on, the subsequent formation of polymers.

### Additive-mediated stabilization of α_1_-antitrypsin against intermediate formation

Osmolytes exert non-specific global effects on protein integrity, primarily by altering solvent behaviour or through unfavourable interactions with the protein backbone, favouring a compact folded state [58]. For α_1_-antitrypsin and neuroserpin, the result is a reduced rate of polymerization and an increase in observed *T*_m_ values [33–35]. Considering this general trend and the nature of the unfolding transition measured using CD, it has been suggested that this effect is elicited by stabilization of the serpin native state [34,35]. We set out to use this system to establish the baseline relationship between generic changes in native state stability, reflected partly in the value of *T*_m_, and the rate of α_1_-antitrypsin intermolecular association. TMAO was used due to its marked stabilization of α_1_-antitrypsin at high concentrations [35], as well as representatives of two other classes of osmolyte, sucrose (a polyol) and sodium sulfate (a kosmotropic salt). AT_C232S_, in which the free cysteine is mutated to a serine, was the background used for later mutagenesis and so was used in subsequent experiments.

**Figure 2:**
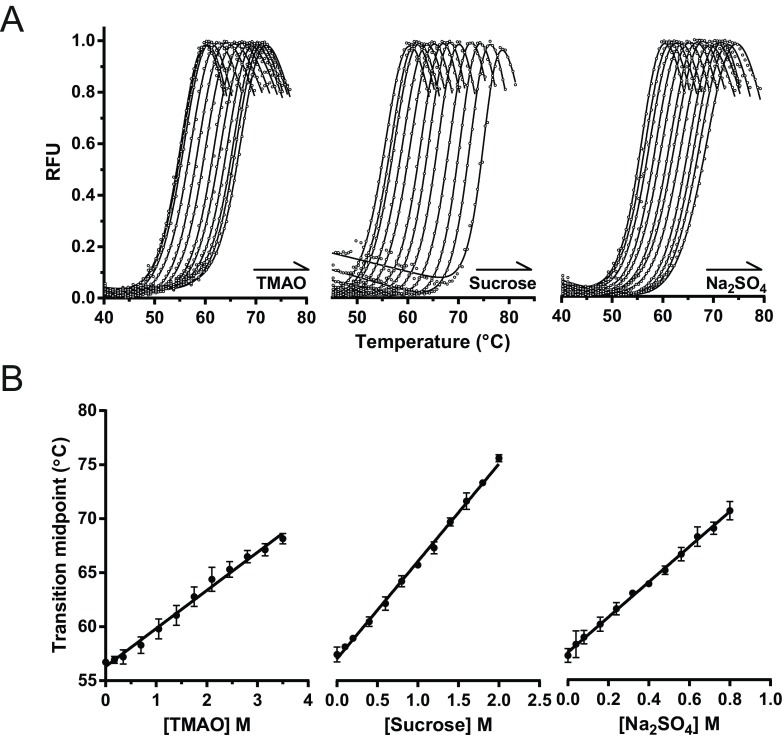
The effect of TMAO, sucrose and sodium sulfate on thermal transition midpoints of α_1_-antitrypsin AT_C232S_, at a concentration of 0.1 mg/ml, was heated at a rate of 1°C/min from 25°C to 95°C in a buffer containing 25 mM NaH_2_PO_4_/Na_2_HPO_4_, 75 mM NaCl (pH 7.4) and 5× SYPRO Orange in the presence of 0–3.5 M TMAO (left-hand panels), 0–2 M sucrose (middle panels) and 0–0.8 M sodium sulfate (right-hand panels). (**A**) The resulting normalized curves of fluorescence of SYPRO Orange, calculated from the mean of four independent experiments, were fitted to an equation describing two-state unfolding, with progressive stabilization evident at increasing additive concentrations. (**B**) The midpoint temperatures showed a linear dependence on additive concentration. Error bars represent the S.D. for four independent measurements.

Thermal unfolding experiments of AT_C232S_ were conducted in the presence of the SYPRO Orange reporter dye. Owing to our observation of the kinetic-biased nature of the unfolding curves, we did not directly infer thermodynamic parameters from them. Several TMAO concentrations between 0 and 3.5 M (Figure 2A, left-hand panel), 0 to 2 M sucrose (Figure 2A, middle panel) and 0 to 0.8 M sodium sulfate (Figure 2A, right-hand panel) were used. As expected, there was a progressive shift of the melting curve to higher transition temperatures in the presence of higher levels of TMAO. Despite their different chemical properties, the other two stabilizing compounds showed a qualitatively similar effect on α_1_-antitrypsin stability, albeit at substantially different concentrations. The unfolding curves were accommodated well by a two-state equation, from which *T*_m_ values could be calculated. For all three additives, the *T*_m_ values showed a clear linear dependence on osmolyte concentration (Figure 2B), consistent with increasing stability of the native state against formation of a partially unfolded state. The stabilization by TMAO, sucrose and sodium sulfate was 3.5±0.2°C/M, 9.0±0.1°C/M and 16.3±0.3°C/M respectively.

### The effect of stabilizing compounds on polymerization half-time

For the determination of the rate of polymerization, a FRET-based approach was used to monitor intermolecular association. This assay uses nitrilotriacetic acid-conjugated fluorescent dyes that bind to an N-terminal His_6_ affinity tag and report an increase in proximity between interacting monomers through an increase in FRET efficiency [36]. The polymerization of AT_C232S_ was followed in the presence of the same range of concentrations of co-solute as detailed above, at 55°C, 60°C and 65°C with 0.2 mg/ml protein and 4 μM reporter dye. The resulting progress curves were well described by a two-phase exponential association function. Although convenient for subsequent analyses, this concordance is not expected to be a true reflection of the polymerization mechanism; in particular, it does not explain the presence of a concentration-dependent behaviour [28,36]. We preferred the use of the polymerization half-time (*t*_0.5_) as a simple descriptor for the curves, in order to avoid assumptions about the reaction pathway inherent in more complex treatments. Additionally, this facilitated comparison with other studies, as polymerization rates have typically been reported in the literature either as half-times or first-order rate constants, such as *k*_obs_, which are related to one another by the simple transformation *t*_0.5_=0.693/*k*_obs_. The parameterized curves were therefore subsequently used for the numerical determination of the half-time of the polymerization. Indicative values are presented in Table 2.

**Table 2:** Representative polymerization half-lives in the presence of different solutes AT_C232S_ was heated at a constant temperature in the presence of several different concentrations of stabilizing additives, and polymerization was monitored by FRET. The time taken to reach a half-maximal signal±S.D. for a subset of these experiments is shown.

	Temperature					
	55°C		60°C		65°C	
Additive	Concentration (M)	*t*_0.5_×10^−2^ s	Concentration (M)	*t*_0.5_×10^−2^ s	Concentration (M)	*t*_0.5_×10^−2^ s
None	–	4.28±0.13	–	0.60±0.06	–	0.23±0.02
TMAO	2.9	97.9±6.0	3.5	19.2±0.6	3.5	4.7±0.1
Sucrose	1.1	64.2±27.0	1.7	119.0±16.3	2	67.3±5.3
Sodium sulfate	0.8	180.1±17.0	0.8	71.7±2.8	0.8	11.9±4.1

### The relationship between rates of polymerization and *T*_m_ values

**Figure 3:**
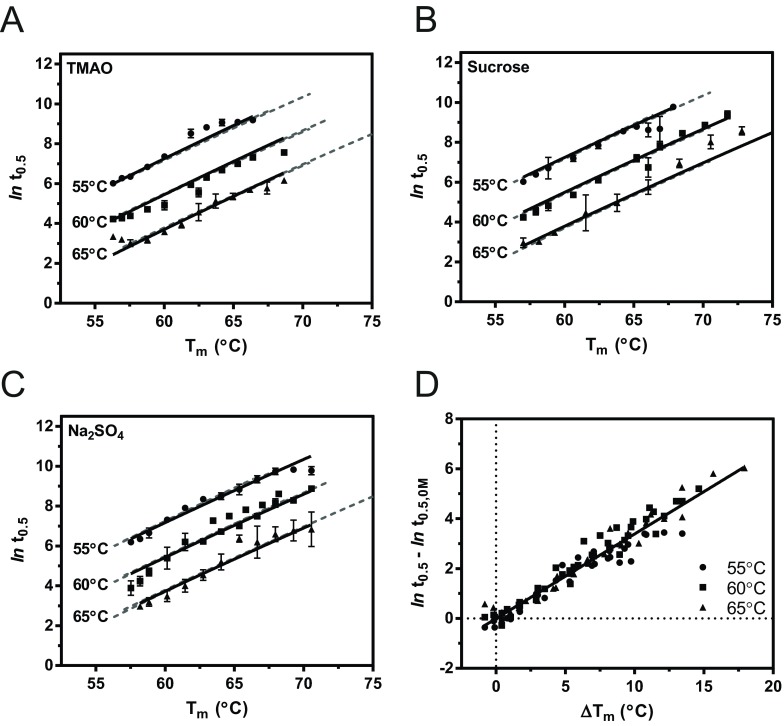
The rate of polymerization in the presence of additives Polymerization was monitored using a FRET-based assay for AT_C232S_ in the presence of (**A**) TMAO, (**B**) sucrose and (**C**) sodium sulfate at 55°C, 60°C and 65°C. Additive concentration was converted into *T*_m_ values based on the linear relationships shown in Figure 2, permitting direct comparison of the three additives on a common scale (abscissa). The half-time of polymerization, *t*_0.5_, in the presence of the osmolytes, was obtained by fitting progress curves to a two-phase exponential equation for numerical derivation of the time at which the signal reached 50% of the range of the experiment. The data at all temperatures were globally fit to an equation describing the effect of experimental temperature, *T*_m_ and the activation energy of an irreversible step, on the rate of polymerization [56,59]. For comparison, the results of the regression with other co-solutes are shown as broken lines. (**D**) The half-times for all co-solutes (*t*_0.5_) were normalized by subtracting the natural logarithm of the value for the AT_C232S_ control (*t*_0.5,0M_) at each temperature, resulting in a superimposition of all data presented in (**A**–**C**). These combined data were used to perform a linear regression analysis, as described in the text. Results are means±S.E.M. from at least four independent experiments.

To compare the effect of the co-solutes on polymerization, the linear relationship between stabilizer concentration and *T*_m_ value (Figure 2B) was used to represent the data on a common scale. As the concentration of each co-solute increased, so too did the resulting values of *T*_m_ and *t*_0.5_, and the natural logarithm of the *t*_0.5_ values showed a strong positive dependence on *T*_m_ (Figures 3A–3C). We had described previously an Arrhenius-type temperature dependence for polymerization [36], and had found the thermal unfolding pathway to reflect a reversible transition followed by a subsequent rate-limiting irreversible process. We were therefore able to globally fit each multiple-temperature dataset to an equation that describes a kinetically controlled (non-equilibrium) process [56,59]:

(1) $$t_{0.5,\mathrm{stab}}\;=0.693/\exp^{\left(-E_{\mathrm{act},\mathrm{app}}/R\left(1/T-1/T_{\mathrm{stab}}^{⋆}\right)\right)}$$

where *E*_act,app_ is the apparent activation energy for the polymerization reaction, *R* is the universal gas constant, *T* is the experimental temperature in K, and T*_stab_ is a reference temperature at which the polymerization rate of the stabilized preparation is 1 s^−1^, derived from the apparent *T*_m_ value by T*_stab_=*aT*_m_, where *a* is a scaling factor that is optimized during curve fitting.

Despite marked chemical differences between these compounds, the resulting curves for TMAO, sucrose and sodium sulfate reflected a remarkable correspondence (Figures 3A–3C, broken lines). Deviations occurred essentially at the highest concentrations, where the distinct chemical natures of the compounds are more likely to exert an effect. The optimized values of *E*_act,app_ and *a* were correspondingly almost identical for each compound (Supplementary Table S1 at http://www.biochemj.org/bj/460/bj4600103add.htm); all data were therefore combined, with a global parameter optimization yielding values of 320±4 kJ/mol and 1.05 for *E*_act,app_ and *a* respectively.

This Arrhenius-type behaviour could also be exploited to normalize the data collected at different temperatures; subtraction of the ln(*t*_0.5_) value obtained in the absence of co-solute at each experimental temperature (*t*_0.5,0M_) revealed an excellent overlap between the data collected at all three temperatures (Figure 3D). For the sake of comparison with other studies, the results of the thermal denaturation assay were calculated as Δ*T*_m_ values, relative to that of protein in the absence of co-solute. Linear regression of the combined data for the three osmolytes at the three different temperatures yielded the relationship:

(2) $$\ln\left(t_{0.5,\mathrm{stab}}\right)-\ln\left(t_{0.5,0M}\right)=0.34\left(\pm 0.1\right)\;\times \;\Delta T_{m}$$

with *t*_0.5,0M_ representing the half-time and *T*_m,0M_ representing the midpoint of thermal denaturation in the absence of co-solute. This represents an approximation of eqn (1), which, despite describing a hyperbolic relationship, is essentially linear in nature over the range of temperatures considered (Figures 3A–3C). If we compare the natural logarithm of *t*_0.5,stab_ of a stabilized preparation, with respect to that of the control, *t*_0.5,0M_, the result is independent of experimental temperature *T*. It can be shown that, from eqn (1):

(3) $$\ln\left(t_{0.5,\mathrm{stab}}\right)-\ln\left(t_{0.5,0M}\right)\;\approx \;\left(E_{\mathrm{act},\mathrm{app}}\Delta T_{m}\right)/\left(aRT_{m,0M}{}^{2}\right)$$

and, substituting the values determined above, *E*_act,app_/(*aRT*_m,__0M_^2^)=0.34 (see the Supplementary Online Data for details at http://www.biochemj.org/bj/460/bj4600103add.htm). It would be interesting to see whether this form of analysis can be applied to other serpins by substitution of the relevant parameters.

Thus for the non-specific stabilization elicited by these compounds, there is a strikingly direct correspondence between thermal stability, as reported by the thermal denaturation assay, and polymerization kinetics.

### Stabilized α_1_-antitrypsin variants

**Figure 4:**
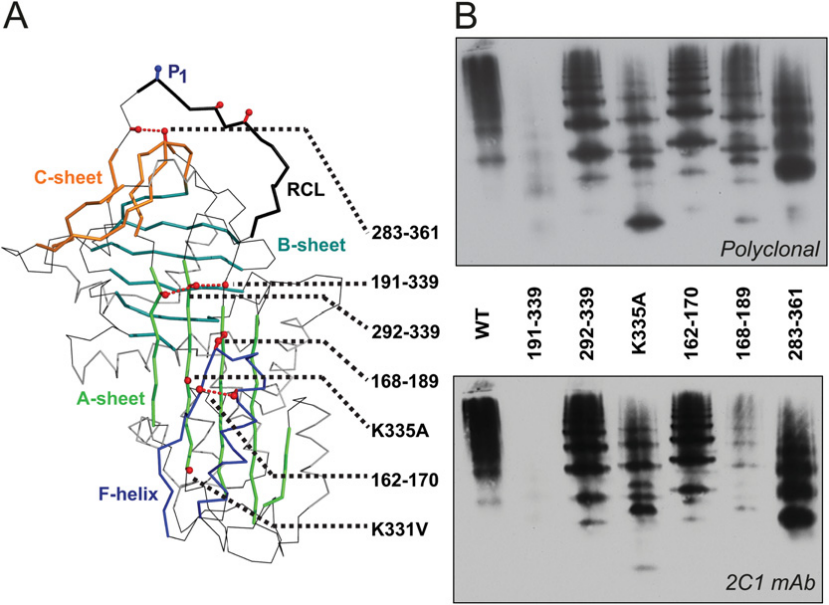
Disulfide-stabilized variants of α_1_-antitrypsin (**A**) The location of stabilizing mutations characterized in the present study are indicated against a cartoon representation of AT_C232S_ α_1_-antitrypsin (prepared using PyMOL (http:://www.pymol.org) and PDB code 1QLP [62]). (**B**) AT_C232S_ and mutants were heated at 60°C for 8 h and separated by non-denaturing PAGE. Western blot analysis was performed using the 2C1 anti-pathogenic polymer antibody (lower panel) before stripping and re-probing with a total α_1_-antitrypsin antibody (upper panel).

Osmolytes exert non-specific global effects on protein integrity primarily through manipulation of solvent behaviour or through unfavourable interactions with the protein backbone, promoting a compact folded state [58]. As mutations affect protein behaviour directly, we sought to determine whether known, and novel, mutations result in a comparable relationship between stability and the rate of polymer formation. Previously, destabilizing mutations in the shutter domain and proximal and distal hinges of the RCL have been found to increase the rate of polymerization [28]. Further, disulfides that restrict the movement of mobile elements of the serpin scaffold have been found to interfere with polymerization [21,22,30,31,60]. It is evident that, in some cases, this interference manifests as reduction in, rather than loss of, the ability to polymerize [22,31]. Seven variants were considered (Figure 4A): a mutant to prevent the release of strand 1C from the C-sheet (283–361) [30]; a mutant to prevent release of strand 5A from the A-sheet (292–339) [21]; a mutant to stabilize the C-terminus of the F-helix (162–170); a mutant to prevent opening of β-sheet A (191–339); a mutant of the disulfide tethering the F-helix to strand 3A (168–189) [31]; and two thermostable point mutants (K335A and K331V) [29,39].

Serpins rely on differences in the energy of two conformational states during the inhibition of a target protease, and residual inhibitory activity provides an alternative function-specific measure of native stability relative to the inserted six-stranded form of the protein (Table 3). As RCL insertion is an obligate component of the inhibitory mechanism, 191–339 was essentially non-inhibitory; its SI was consistent with approximately 4% of the material lacking the disulfide bond. Activity against bovine α-chymotrypsin was reduced in the K331V and 168–189 forms, but was similar to the AT_C232S_ control for the other variants. All variants showed moderate to pronounced enhancement of thermostability in a SYPRO-based thermal melt assay (Table 3).

**Table 3:** Biophysical and biochemical characteristics of recombinant wild-type and mutants of α_1_-antitrypsin Variants were assessed for their midpoint of denaturation, stoichiometry of inhibition (SI) and association rate constant (*k*_ass_) against bovine α-chymotrypsin. The results are the mean of at least three independent experiments. Rows labelled ‘reduced’ are for protein treated with 100 mM 2-mercaptoethanol.

Variant	*T*_m_ (°C)*	SI†	*k*_ass_ (M^−1^·s^−1^)	*k*_ass_×SI
AT_C232S_	55.0	1.1±0.02	1.5±0.2×10^6^	1.5×10^6^
Disulfide mutants				
162–170	64.5	1.0±0.02	1.6±0.3×10^6^	1.6×10^6^
162–170 (reduced)	55.0	1.0±0.05	1.5±0.2×10^6^	1.5×10^6^
168–189	67.5	8.5±0.4	4.2±0.7×10^3^	3.6×10^4^
168–189 (reduced)	56.0	1.2±0.03	1.0±0.1×10^6^	1.2×10^6^
191–339	59.5	27±1.4	2.0±0.4×10^4^	5.4×10^5^
191–339 (reduced)	55.0	1.0±0.03	0.9±0.04×10^5^	0.9×10^5^
283–361	60.5	1.0±0.01	1.3±0.1×10^6^	1.3×10^6^
283–361 (reduced)	56.5	1.0±0.04	1.4±0.6×10^6^	1.4×10^6^
292–339	61.5	1.0±0.03	1.4±0.3×10^6^	1.4×10^6^
292–339 (reduced)	57.0	1.0±0.03	1.7±0.04×10^6^	1.7×10^6^
Strand 5A/F-helix				
K331V	59.0	3.9±0.2	1.0±0.1×10^6^	3.9×10^6^
K335A	70.0	1.0±0.02	0.9±0.1×10^6^	0.9×10^6^

*All standard errors were less than precision of the technique on the instrument used (±0.5°C).

†Standard errors were calculated by regression of a transformed linear equation with the intercept at the abscissa as a parameter.

### Polymers recognized by the 2C1 monoclonal antibody can form in the presence of stabilizing disulfide bonds

Preliminary experiments revealed that 283–361 and 292–339 were able to polymerize, despite previously published observations that they were incapable of doing so [21,30]. Polymerization can occur by more than one pathway; conditions such as the use of heat, chemical denaturants and mutations can consequently alter the configurations of the resulting polymer [22,27]. We therefore made use of the 2C1 antibody, which provides a means to identify α_1_-antitrypsin polymers generated *in vitro* that are non-orthologous to those isolated from patients [26,27]. The α_1_-antitrypsin variants were heated at 60°C for 8 h, the polymers separated by non-denaturing PAGE, and then visualized by Western blotting. After heating, all variants had predominantly formed higher molecular mass species, with the exception of 191–339, which due to an absence of detectable material, had most probably precipitated from solution (Figure 4B, upper panel). In order to determine whether any mutations altered the polymerization pathway, we compared recognition of the mutants with the 2C1 antibody. AT_C232S_, 292–339, 283–361, 162–170 and K335A formed 2C1^+^ polymers at 60°C (Figure 4B, lower panel). In contrast, the 168–189 variant produced predominantly 2C1^−^ polymers, suggesting that it causes the reaction to favour a different pathway. Hence four of these mutants failed to prevent any structural rearrangement prerequisite to the polymerization mechanism. As disulfide bonds can result in an overall stabilization of the native fold by reducing local secondary structure mobility [32], we sought to determine whether these mutants affect polymerization through specific or non-specific effects.

### The effect of stabilizing mutations on the rate of polymerization

**Figure 5:**
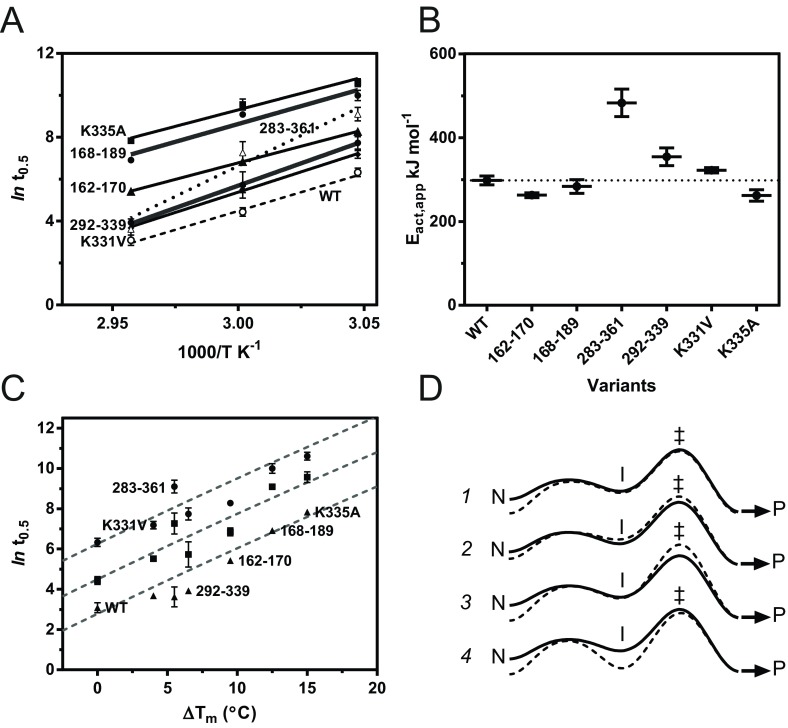
Relationship between mutant thermal stability and the rate of polymerization Polymerization of AT_C232S_ and the stabilized mutants was monitored using the FRET-based assay. (**A**) The relationship between the natural logarithm of the half-times, and the inverse absolute temperature at which they were determined, is presented. Based on the Arrhenius-type temperature dependence, linear regression analysis permitted the derivation of *E*_act_ values. The AT_C232S_ control is shown as a dashed line; in comparison, the 283–361 mutant shows a markedly different behaviour (dotted line). WT, wild-type. (**B**) A summary of the calculated activation energy values (*E*_act,app_) is shown. The dotted line corresponds to the value calculated for the AT_C232S_ control; 283–361 and 292–339 were found to be significantly different from the control (*P*<0.001 and *P*<0.05 respectively) by one-way ANOVA with Bonferroni correction. (**C**) Half-times of polymerization at 55°C (●), 60°C (■) and 65°C (▲) are shown in relation to the *T*_m_ values of the variants. The dashed lines represent the dependence of polymerization on experimental temperature and *T*_m_ in the presence of osmolytes, using the data presented in Figure 3. (**D**) Notional energy landscapes of the progression from the native conformation, N, to the partially unfolded intermediate, I, via the transition state of the irreversible step, ‡, culminating in polymer formation, P. The solid line represents the ‘wild-type’ scenario, whereas the broken line indicates possible alternate changes to the pathway resulting in a reduction in polymer formation. Results are means±S.E.M.

Using the FRET-based assay, the times taken to reach half-maximal polymerization signal were determined for AT_C232S_ and stabilized variants at 55°C, 60°C and 65°C. The results are shown in Table 4. At 55°C, all of the mutants considered demonstrated a 1.5- to 25-fold delay with respect to the AT_C232S_ control, whereas at 65°C 283–361, 292-339 and K331V were comparable with the control. We have previously shown Arrhenius-type behaviour for α_1_-antitrypsin polymerization in the experimental temperature range [36]. When ln(*t*_0.5_) was plotted against the reciprocal absolute temperature, this behaviour was found to hold for these stabilized mutants as well (Figure 5A). Activation energies obtained from the resulting slopes revealed that, despite conferral of a marked increase in thermal stability, all but two of the mutants failed to significantly alter the kinetic barrier to polymer formation (Figure 5B and Table 4), including the most polymer-resistant variant K335A. This serves to highlight that resistance to polymerization does not of itself indicate interference with the irreversible (and thus kinetically determined) component of the pathway.

**Table 4:** Polymerization of the α_1_-antitrypsin variants The times taken to reach a half-maximal polymerization FRET signal (in s) at the different assay temperatures are shown, with the standard error calculated from at least five independent experiments, along with the calculated apparent activation energy of the reaction. The *P* values for mutants with a significantly different *E*_act_ value to wild-type (based on a multiple one-way ANOVA comparison with AT_C232S_ using Bonferroni's correction) are indicated. n.s, not significant.

	Temperature				
Variant	55°C	60°C	65°C	*E*_act_ (kJ/mol)	Significance
AT_C232S_	880±127	109±9	38±7	298±10	–
162–170	3.93 (±0.09)×10^3^	941±41	226±7	262±6	n.s.
168–189	21.9 (±1.7)×10^3^	8.87 (±0.34)×10^3^	1.01 (±0.05)×10^3^	283±16	n.s.
283–361	8.97 (±0.96)×10^3^	1.43 (±0.23)×10^3^	37±8	482±32	*P*<0.001
292–339	2.29 (±0.20)×10^3^	307±56	51±3	354±21	*P*<0.05
K331V	1.31 (±0.07)×10^3^	248±9	39±1	322±6	n.s.
K335A	40.4 (±3.2)×10^3^	14.2 (±1.0)×10^3^	2.52 (±0.10)×10^3^	261±13	n.s.

The collective stability-polymerization profile of the mutants was compared with that determined for the wild-type protein in the presence of osmolytes. As shown in Figure 5(C), there is a reasonable overall correspondence between the two, but with notable deviations. Three types of behaviour are evident.

(i) 168–189, K331V and K335A behave in a similar manner to wild-type protein stabilized by osmolytes. This suggests they have a native state with an increased thermodynamic stability, consistent with conclusions drawn from denaturant-mediated unfolding of the latter two variants [29,50]. However, the relative stability of the thermal unfolding intermediate appears unaffected with respect to the transition state of the kinetically controlled step (Figures 5D, *1*).

(ii) 292–339 and 162–170 increase the overall *t*_0.5_ value, but not to the extent predicted from their Δ*T*_m_ values. This would be consistent with a mode of action in which the native state is only partially stabilized. The thermal unfolding intermediate and kinetic transition states are also destabilized to a slightly greater (292–339) or lesser (162–170) extent (Figure 5D, *2*), as seen by a modest change in *E*_act_ value (Table 4).

(iii) The restraints imposed by the 283–361 mutant present the greatest energetic barrier to polymer formation, with an *E*_act_ value 1.6-fold higher than the control (Table 4). At 55°C and 60°C, 283–361 has a greater *t*_0.5_ value than predicted from the value of Δ*T*_m_ (Figure 5C). Thus it probably both stabilizes the native state and destabilizes the transition state of the irreversible step, increasing the kinetic barrier to polymerization (Figure 5D, *3* and *4*).

Conformational mobility of the RCL is clearly enhanced in the transition state of the irreversible step, as shown by its destabilization in the presence of the 283–361 and 292–339 disulfides. As these mutants are still able to form polymers (Table 4) that are recognized by the 2C1 antibody (Figure 4B), this loss of mobility merely increases the kinetic barrier to polymerization, rather than preventing it altogether. We note that these observations differ from single-temperature studies that concluded an absolute requirement for mobility of the 283–361 and 292–339 regions during polymerization [21,30].

In addition, as the rate of polymerization is strongly coupled to the thermal stability of the α_1_-antitrypsin native state, it is deviations from the expected profile that provide information about effects on irreversible changes along the polymerization pathway. Of the mutants considered in the present study, only 283–361 has a pronounced destabilizing effect on the kinetically controlled step.

### Re-appraisal of published rates of polymerization for mutants of α_1_-antitrypsin

Several studies have previously characterized stabilized and destabilized mutants of α_1_-antitrypsin. For a combined analysis, five published datasets are considered in the present study [28,29,49–51]; when combined with the 60°C data described in the present paper, this represents 36 unique mutants. These datasets differ in the manner in which *T*_m_ value was determined (CD and SYPRO Orange), the *T*_m_ value reported for the wild-type control, the temperature at which polymerization was recorded (45°C, 52°C and 60°C) and the means by which it was monitored (tryptophan fluorescence and non-denaturing PAGE analysis). By way of comparison, a further 12 are considered separately, from a study in which rates were obtained from the change in bis-ANS fluorescence [52], and one in which mutants were intentionally designed to interfere with polymerization but not stability [36]. As shown in the present study, due to the apparent Arrhenius-type dependence of the polymerization rate on temperature, it was possible to normalize the *t*_0.5_ values by reporting them as the absolute difference to the natural logarithm of the wild-type control under the specific conditions of each experiment. Similarly, transition midpoints could be reported as Δ*T*_m_ values relative to the wild-type control.

**Figure 6:**
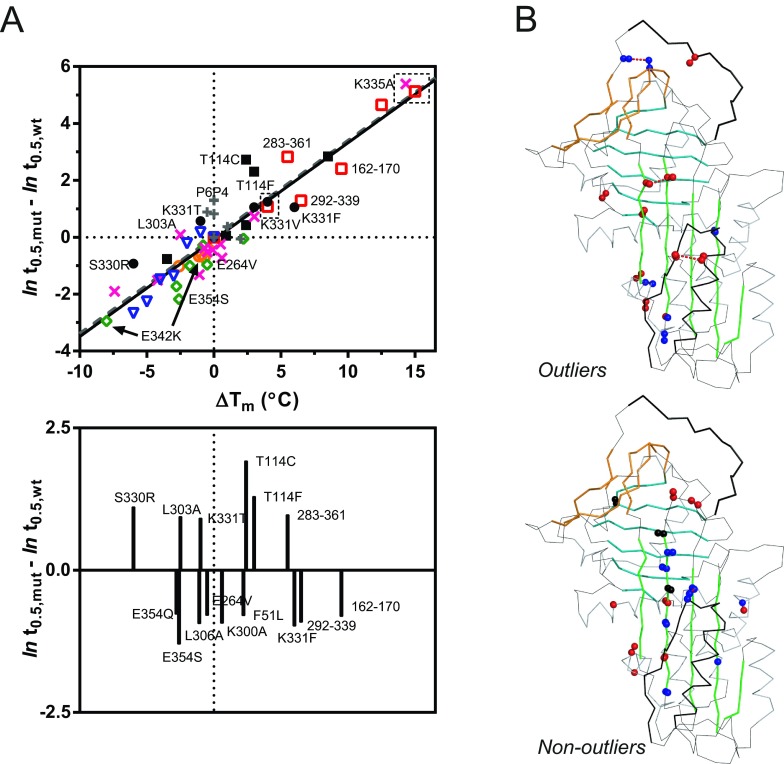
Combined retrospective analysis (**A**) The top panel presents an analysis of the relationship between polymerization kinetics and *T*_m_ value compiled from six studies: Dafforn et al. [28] (green open diamonds), Parfrey et al. [49] (black closed squares), Gilis et al. [29] (black closed circles), Knaupp et al. [51] (orange open circles), Knaupp et al. [50] (pink crosses), and the mutants described in the present study (red open squares). In addition, included by way of comparison, are values determined by Cabrita et al. [52] (blue open triangles) using bis-ANS and Haq et al. [36] (grey crosses) using RCL mutants. The sets of data differed in the manner in which *T*_m_ values were determined, the *T*_m_ reported for the wild-type control and the temperature and means by which polymerization was monitored. The half-times and Δ*T*_m_ values were normalized relative to the values for the wild-type control as determined in each study. The solid line indicates the linear regression performed using the six datasets; the broken line shows the relationship determined using osmolytes (from Figure 3D). The lower panel presents shortlisted mutants for which the magnitude of difference in ln(*t*_0.5_) from the trend line exceeded ±0.75 units. (**B**) The upper panel shows the same select mutants, mapped on to the structure of α_1_-antitrypsin using PyMOL. Blue spheres indicate less ability to polymerize than predicted and red spheres indicate higher rates of polymerization than predicted from Δ*T*_m_. The lower panel indicates mutants for which the rate of polymerization is consistent with the expected value; red spheres denote those for which Δ*T*_m_ is negative (destabilizing) and blue designates a positive Δ*T*_m_ (stabilizing).

The combined result is shown in the upper panel of Figure 6(A). Although there are clear deviations from the overall trend, the Figure highlights that the rate of polymerization is overwhelmingly determined by the thermal stability of the native state for both stabilizing and destabilizing variants. Remarkably, when a linear regression was performed of the data, there was a virtually identical correspondence with the relation-ship between osmolyte-mediated stabilization and rate of polymerization (Figure 6A, upper panel, dashed line).

Of particular interest, is the ability to identify outliers to the curve, shown in the lower panel of Figure 6(A) and mapped on to the structure of α_1_-antitrypsin in Figure 6(B). These are mutants that affect polymerization, either positively or negatively, in a manner that is not completely accounted for by the stability of the native state. In particular, mutants increasing the packing of a ‘pocket’ situated proximal to the F-helix, at Thr^114^ [61], resist polymerization to a greater extent than would be predicted from the model. Conversely, the loss of the salt bridge mediated by Glu^354^ of the RCL promotes polymerization to a greater degree than expected, possibly for the same reason that 283–361 disfavours it. RCL mutants designed to interfere specifically with the polymerization mechanism also show an atypically decreased rate. Although not providing the level of detail that multiple temperature experiments can, the ability to identify outliers to the normalized stability-polymerization curve is useful for pinpointing mutations that affect the stability of non-native components of the pathway.

### Summary

The present study concerns the means by which polymerization is perturbed by mutation: the relative contribution of specific effects on the underlying structural mechanism and non-specific effects on protein stability. The remarkable congruence between additive-induced and mutation-induced effects on polymerization strongly suggests that changes in global stability are the dominant factor.

This result has clear relevance to the characterization of polymer-generating serpins. Although a general trend has been noted in other studies, the extent and consistency of the underlying interrelationship between stability and polymerization has been under-appreciated in the literature. On the basis of the present study, it is evident that in the absence of more refined approaches, an increase in resistance to polymerization is of itself insufficient to support interpretations related to the underlying mechanism. It is suggested that quantification of the deviation from the described correlation, coupled with a multiple-temperature analysis, should be performed to ascertain the degree to which changes in global stability influence the outcome of any such experiment.

## Online data

Supplementary data
