# Supplementary material for: Altered native stability is the dominant basis for susceptibility of α1-antitrypsin mutants to polymerization
Source: Biochem J. 2014 Apr 25;460(Pt 1):103–15. doi: 10.1042/BJ20131650 (PMC4080824; doi:10.1042/BJ20131650)
Supplement: Supplementary data [file bj4600103add.pdf]

BJ Biomolecules

Biochem. J. (2014) 460, 103–115 (Printed in Great Britain) doi:10.1042/BJ20131650

online data

SUPPLEMENTARY ONLINE DATA

Altered native stability is the dominant basis for susceptibility of α_1-antitrypsin mutants to polymerization

James A. IRVING*[1,2], Imran HAQ†[1], Jennifer A. DICKENS*, Sarah V. FAULL* and David A. LOMAS†

*Cambridge Institute for Medical Research, Department of Medicine, University of Cambridge, Wellcome Trust/MRC Building, Hills Road, Cambridge CB2 0XY, U.K.
†Wolfson Institute for Biomedical Research, The Cruciform Building, University College London, Gower Street, London WC1E 6BT, U.K.

Biochemical Journal www.biochemj.org

EXPERIMENTAL

Derivation of eqn (3)

Eqn (2) in the main text reflects the approximately linear dependence of the difference in log half-times on ΔT_{m} value when comparing stabilized and control α_1-antitrypsin preparations. This represents an approximation of eqn (1), which, despite describing a hyperbolic relationship, is almost linear in nature over the range of temperatures considered (Figures 3A–3C of the main text). If we compare the natural logarithm of $t_{0.5,\mathrm{stab}}$ of a stabilized protein with respect to that of the control, $t_{0.5,\mathrm{wt}}$, the result is independent of experimental temperature, T. From eqn (1):

$$\begin{aligned}
&\ln(t_{0.5,\mathrm{stab}}) - \ln(t_{0.5,\mathrm{wt}}) \\
&\quad = (E_{\mathrm{act,app}}/R)\,(1/T - 1/T^*_{\mathrm{stab}}) \\
&\qquad - (E_{\mathrm{act,app}}/R)(1/T - 1/T^*_{\mathrm{wt}}) \\
&\quad = -\,(E_{\mathrm{act,app}}/R)\,(1/T^*_{\mathrm{stab}} - 1/T^*_{\mathrm{wt}}) \\
&\quad = (E_{\mathrm{act,app}}/R)\,(T^*_{\mathrm{stab}} - T^*_{\mathrm{wt}})/(T^*_{\mathrm{stab}}T^*_{\mathrm{wt}}) \qquad \text{(S1)}
\end{aligned}$$

where, as explained in the text, T^* is a reference temperature at which the rate of polymerization is 1 s^{-1}. For a difference $\Delta T^* = T^*_{\mathrm{stab}} - T^*_{\mathrm{wt}}$, it follows that:

$$\ln(t_{0.5,\mathrm{stab}}) - \ln(t_{0.5,\mathrm{wt}}) = (E_{\mathrm{act,app}}\Delta T^*)/\left[RT^*_{\mathrm{wt}}(T^*_{\mathrm{wt}}+\Delta T^*)\right] \qquad \text{(S2)}$$

As T^* values are reported in Kelvin, for the relevant experimentally determined values, $\Delta T^* < 0.1 \times T^*_{\mathrm{wt}}$. At small values of ΔT^* therefore, the equation simplifies to:

$$\ln(t_{0.5,\mathrm{stab}}) - \ln(t_{0.5,\mathrm{wt}}) \approx (E_{\mathrm{act,app}}\Delta T^*)/(RT^{*2}_{\mathrm{wt}}) \qquad \text{(S3)}$$

and if $T^*_{\mathrm{wt}} = aT_{\mathrm{m,wt}}$,

$$\ln(t_{0.5,\mathrm{stab}}) - \ln(t_{0.5,\mathrm{wt}}) \approx (E_{\mathrm{act,app}}\Delta T_{\mathrm{m}})/(aRT_{\mathrm{m,wt}}{}^2) \qquad \text{(S4)}$$

For values $E_{\mathrm{act,app}} = 3.20 \times 10^3$ kJ/mol, $a = 1.05$ and $T_{\mathrm{m,wt}} = 55\,°\mathrm{C}$, calculated from the experimental data. $E_{\mathrm{act,app}}/aRT_{\mathrm{m,wt}}{}^2 = 0.34$, which is identical with the slope of the linear regression calculated from the experimental data and presented in eqn (2).

[1] These authors contributed equally to this work.
[2] To whom correspondence should be addressed at the present address: Wolfson Institute for Biomedical Research, The Cruciform Building, University College London, Gower Street, London WC1E 6BT, U.K. (email j.irving@ucl.ac.uk).

© 2014 The Author(s)
The author(s) has paid for this article to be freely available under the terms of the Creative Commons Attribution Licence (CC-BY) (http://creativecommons.org/licenses/by/3.0/) which permits unrestricted use, distribution and reproduction in any medium, provided the original work is properly cited.

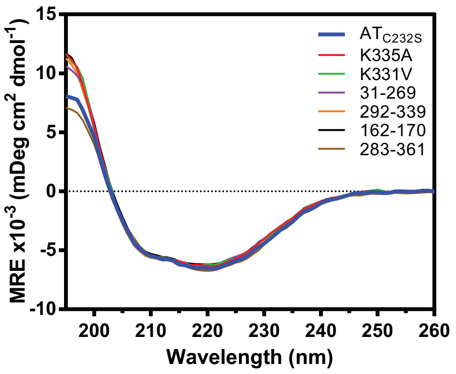

Figure S1 Recombinant mutant CD spectra

The normalized CD spectra of the mutants considered in the present study were recorded between 260 nm and 190 nm in 10 mM Na_2HPO_4/NaH_2PO_4 (pH 7.4) buffer, and show a similar shape to that of the wild-type protein (blue).

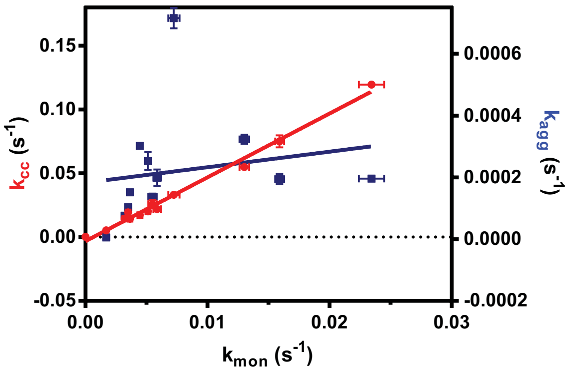

Figure S2 Comparison of polymerization parameters determined by non-denaturing PAGE and bis-ANS fluorescence

All values are taken from Knaupp et al. [1]. The reported rate of disappearance of monomeric α_1-antitrypsin evaluated by non-denaturing PAGE (k_{mon}) is shown in comparison with the initial rapid rate of increase in bis-ANS fluorescence (k_{cc}; red) and subsequent slow decrease (k_{agg}; blue) for several α_1-antitrypsin variants. There is a strong linear correspondence ($R^2 = 0.99$) between gel densitometry and the rapid initial increase in bis-ANS fluorescence, but not between gel densitometry and the slow phase ($R^2 = 0.03$).

Table S1 Optimal parameters obtained from the non-linear regression analysis of T_m and polymerization half-time

Values were calculated for the apparent activation energy $E_{act,app}$ and scaling factor a in the presence of TMAO, sucrose and sodium sulfate. Standard errors of a were <1 %.

Additive	$E_{act,app}$ (kJ/mol)	a
All (combined)	320 ± 4	1.05
TMAO	328 ± 6	1.05
Sucrose	318 ± 8	1.05
Sodium sulfate	317 ± 7	1.05

© 2014 The Author(s)
The author(s) has paid for this article to be freely available under the terms of the Creative Commons Attribution Licence (CC-BY) (http://creativecommons.org/licenses/by/3.0/) which permits unrestricted use, distribution and reproduction in any medium, provided the original work is properly cited.

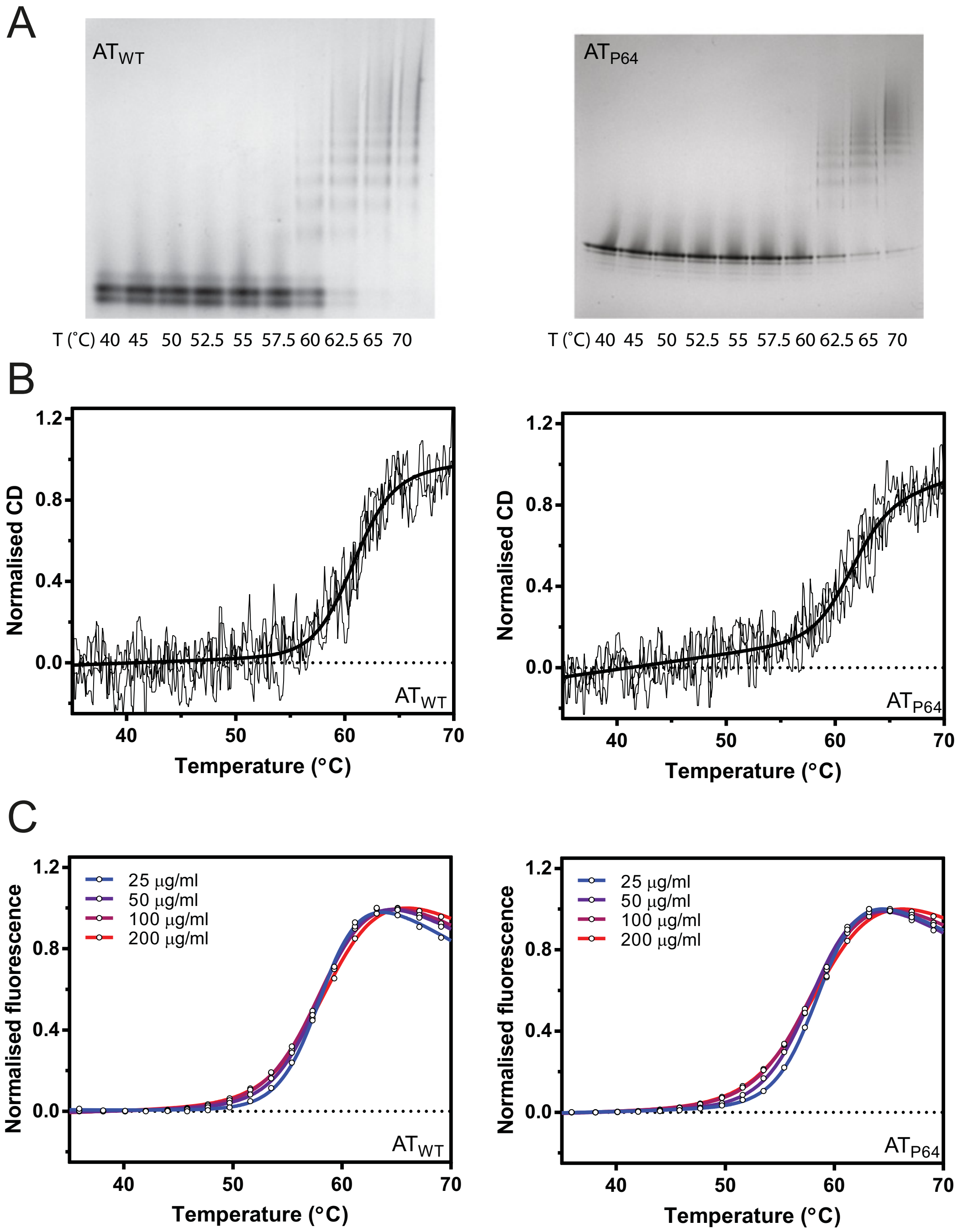

Figure S3 Processes forming the basis of thermal unfolding profiles

(**A**) A typical non-denaturing PAGE profile, with aliquots of recombinant α_1-antitrypsin (left-hand panel) removed over the course of a CD-based thermal unfolding experiment. The concentration was 0.1 mg/ml and the sample was heated at 1 °C/min; the prevailing sample temperature is indicated. The gel was visualized using Coomassie Blue. The same experiment performed using the less polymerization-prone AT_{P64} variant is shown in the right-hand panel. (**B**) The fit of the two-state unfolding curves, reported in Figure 1(E) of the main text, to normalized CD data read at 225 nm, for AT_{WT} (left-hand panel) and AT_{P64} (right-hand panel). The temperature was increased at a rate of 5 °C/min and the protein concentration was 0.025 mg/ml. Data from three independent experiments are shown. (**C**) Thermal unfolding profiles from two independent experiments, measured as a function of increase in SYPRO Orange fluorescence of AT_{WT} (left-hand panel) and AT_{P64} (right-hand panel). Samples at 0.025, 0.05, 0.1 and 0.2 mg/ml were heated at a rate of 5 °C/min, with slight variation of profile shape, but almost identical transition midpoints.

© 2014 The Author(s)
The author(s) has paid for this article to be freely available under the terms of the Creative Commons Attribution Licence (CC-BY) (http://creativecommons.org/licenses/by/3.0/) which permits unrestricted use, distribution and reproduction in any medium, provided the original work is properly cited.

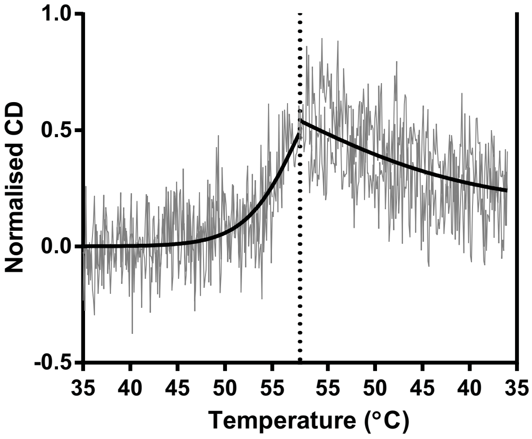

Figure S4 Partial reversibility of temperature-induced change in CD at 225 nm

Recombinant α_1-antitrypsin, at a concentration of 0.1 mg/ml, was heated at a rate of 1 °C/min to 58 °C, approximately the transition midpoint, and cooled at the same rate. The profile shows partial recovery of the initial CD value. Sample temperature was monitored directly using a submerged thermistor probe. Data are from two independent experiments.

REFERENCE

1 Knaupp, A. S., Keleher, S., Yang, L., Dai, W., Bottomley, S. P. and Pearce, M. C. (2013) The roles of helix I and strand 5A in the folding, function and misfolding of α_1-antitrypsin. PLoS ONE **8**, e54766

Received 16 December 2013/13 February 2014; accepted 20 February 2014
Published as BJ Immediate Publication 20 February 2014, doi:10.1042/BJ20131650

© 2014 The Author(s)
The author(s) has paid for this article to be freely available under the terms of the Creative Commons Attribution Licence (CC-BY) (http://creativecommons.org/licenses/by/3.0/) which permits unrestricted use, distribution and reproduction in any medium, provided the original work is properly cited.
